# Reversible Hyperpigmentation in a Patient With Vitamin B12 Deficiency

**DOI:** 10.7759/cureus.63311

**Published:** 2024-06-27

**Authors:** Abdul-Subulr Yakubu, Dzifa Ahadzi

**Affiliations:** 1 Internal Medicine, Tamale Teaching Hospital, Tamale, GHA

**Keywords:** vitamin b12 deficiency symptoms, cobalamin deficiency, pernicious anaemia, vitamin b12, cutaneous hyperpigmentation

## Abstract

Hyperpigmentation is a recognized sign of vitamin B12 deficiency that resolves after vitamin repletion. We present the case of a 58-year-old female with neuropsychiatric symptoms who developed progressive darkening of her hands and feet. A diagnosis of vitamin B12 deficiency secondary to pernicious anemia was made and her symptoms and hyperpigmentation resolved following vitamin repletion. Clinicians should consider vitamin B12 deficiency in the differential diagnosis of palmoplantar hyperpigmentation, as early treatment can avert permanent disability in these patients.

## Introduction

The clinical manifestations of vitamin B12 deficiency are varied and non-specific but commonly include hematologic, neuropsychiatric, and dermatologic abnormalities. Delays in diagnosis and treatment can result in severe complications, including permanent neurologic damage [1]. Hyperpigmentation represents the commonest cutaneous manifestations of vitamin B12 deficiency, and its recognition could aid in early diagnosis and treatment [2]. The following case presentation highlights the multiple ways that vitamin B12 deficiency may present and serves as a reminder to clinicians to consider vitamin B12 deficiency in patients presenting with hyperpigmentation.

## Case presentation

A 58-year-old female presented with progressive darkening of her hands and feet over one year. She has had paresthesia in her feet and an unsteady gait for the past two years, which has worsened, resulting in an inability to walk unsupported. A week before her presentation, she developed a painful right leg swelling following long-distance travel. Her medical history was significant for type 2 diabetes mellitus. She was also diagnosed with severe depression with psychotic symptoms two years ago. Her medications included fluoxetine, olanzapine, metformin, atorvastatin, and omeprazole. She did not smoke or drink alcohol. She was not a vegetarian.

**Figure 1:**
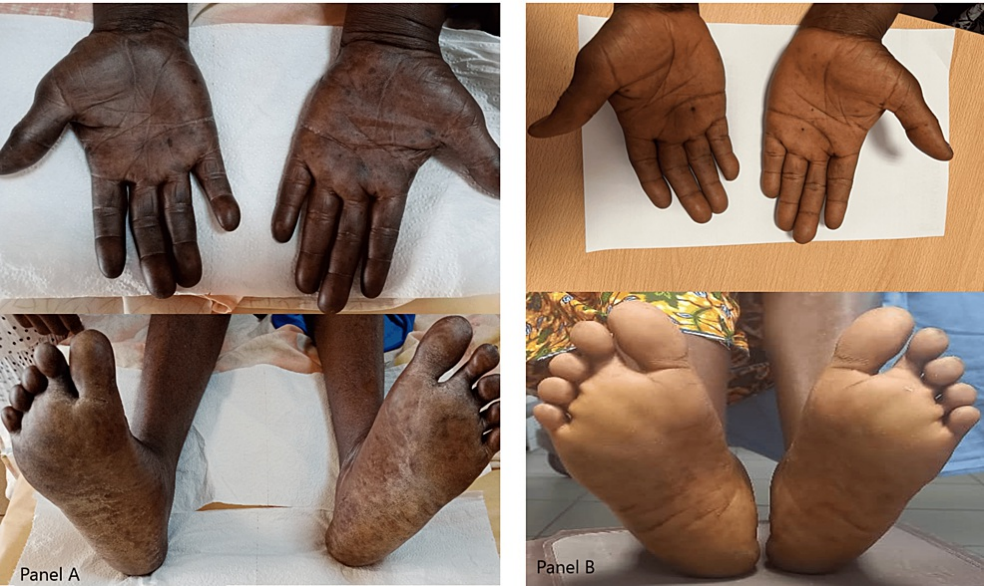
Hyperpigmentation of the patient’s hands and feet at presentation (Panel A) and after three months of treatment with vitamin B12 (Panel B)

On examination, her body mass index was 23.4 kg/m^2^. She had hyperpigmentation of her hands and feet involving the palms and soles (Figure 1A). Her right leg was mildly swollen and tender with differential warmth. Her gait was ataxic, and Romberg’s sign was positive. Muscle power was mildly reduced in the lower limbs.

Laboratory findings were significant for normocytic anemia and reduced serum vitamin B12 levels (34 pmol/l; reference range 133-675 pmol/l). Antibodies to intrinsic factor was strongly positive (213.00 AU/mL; reference <1.20 AU/mL). Serum folate levels were normal (21.0 nmol/l; reference range 6.0-28.0 nmol/l), and she tested negative for human immunodeficiency virus. A Doppler ultrasound scan of the right leg showed deep vein thrombosis (DVT) of the right common femoral, superficial femoral, and popliteal veins.

Anticoagulation with rivaroxaban was initiated for the DVT and continued for six months. A diagnosis of vitamin B12 deficiency secondary to pernicious anemia was also made. Intramuscular vitamin B12 (cyanocobalamin) was initiated at 1 mg daily for seven days, 1 mg weekly for 4 weeks, and then 1 mg monthly. After three months, the pigmentation in her hands and feet had improved (Figure 1B).

**Figure 2:**
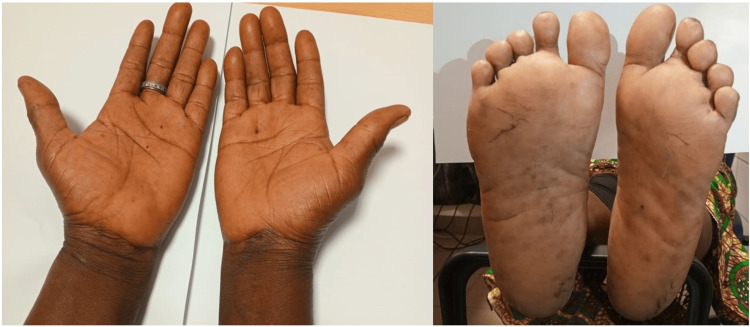
Resolution of pigmentation at the 20-month follow-up

Her mood and gait improved, and she was walking unsupported. The hematologic parameters had normalized, and serum vitamin B12 levels were within normal limits (356 pmol/l). Monthly intramuscular vitamin B12 was continued indefinitely. At follow-up 20 months after the initial presentation, she was well and asymptomatic and the pigmentation in her hands and feet had resolved (Figure 2). Her antidepressant and antipsychotic medications were successfully discontinued, and her glycemic control remained good on metformin.

## Discussion

Vitamin B12 is a water-soluble vitamin derived mainly from animal food sources and is important for hematopoiesis and neuronal function [3]. It is essential for the methylation reactions related to DNA and cell metabolism [1]. Hyperpigmentation has been recognized as a sign of vitamin B12 deficiency and may be the first sign of such a deficient state [2,4-6]. The hyperpigmentation may be generalized, with accentuation in flexural areas, palms, soles, and the oral cavity [5]. Other cutaneous manifestations of vitamin B12 deficiency include hair and nail changes, glossitis, angular stomatitis, and vitiligo [2,7]. The mechanism of hyperpigmentation in vitamin B12 deficiency is believed to be related to increased tyrosinase activity and consequent increased melanin synthesis [8-11]. Vitamin B12 deficiency has also been associated with thrombotic events related to elevated homocysteine levels and may have contributed to the right leg DVT in our patient [12].

Macrocytic anemia is typically seen in vitamin B12 deficiency. The patient presented in this report had normocytic anemia, which could be due to co-existent nutrient/iron deficiency. Vitamin B12 deficiency can, however, manifest without the typical hematologic changes and a normal mean corpuscular volume does not exclude vitamin B12 deficiency [13]. Serum vitamin B12 levels should be assessed regardless of the blood count picture if a deficient state is suspected.

Vitamin B12 deficiency is commonly due to decreased intake, decreased absorption, or the result of interfering medications such as metformin or proton pump inhibitors [14]. Metformin was continued in the patient in this case since she was receiving parenteral B12 replacement with normalized serum levels. This case highlights the diagnostic delays that characterize vitamin B12 deficiency, especially in resource-constrained settings. The presence of neuropsychiatric symptoms, hematologic abnormalities, gastrointestinal features, or dermatologic changes should raise the suspicion of vitamin B12 deficiency, especially in patients with risk factors [3,14].

## Conclusions

The clinical manifestations of vitamin B12 deficiency can be nonspecific, resulting in delayed diagnosis. The diagnosis should be considered in patients with neuropsychiatric, gastrointestinal, hematologic, or cutaneous abnormalities. Vitamin B12 deficiency can manifest without the typical hematologic changes. Hyperpigmentation may be the first sign of vitamin B12 deficiency. It should be considered by clinicians in the differential diagnosis of palmoplantar hyperpigmentation, as early treatment can avert permanent disability in these patients.

## References

[1] Shipton MJ, Thachil J (2015). Vitamin B12 deficiency - a 21st century perspective. Clin Med (Lond).

[2] Brescoll J, Daveluy S (2015). A review of vitamin B12 in dermatology. Am J Clin Dermatol.

[3] Stabler SP (2013). Clinical practice. Vitamin B12 deficiency. N Engl J Med.

[4] Kuenyefu Awindaogo RA, Ekem I, Awuku NA, Salia S, Agyei M, Nartey YA, Awuku YA (2020). Reversible hyperpigmentation in Vitamin B12 deficiency: an Addisonian mimic in clinical practice. PAMJ Clin Med.

[5] Aşkın Ö, Uzunçakmak TKÜ, Altunkalem N, Tüzün Y (2021). Vitamin deficiencies/hypervitaminosis and the skin. Clin Dermatol.

[6] Baker S, Ignatius M, Johnson S, Vaish S (1963). Hyperpigmentation of skin. A sign of vitamin-B12 deficiency. Br Med J.

[7] Kannan R, Ng MJM (2008). Cutaneous lesions and vitamin B12 deficiency: an often-forgotten link. Can Fam Physician.

[8] Agrawala RK, Sahoo SK, Choudhury AK, Mohanty BK, Baliarsinha AK (2013). Pigmentation in vitamin B12 deficiency masquerading Addison's pigmentation: a rare presentation. Indian J Endocrinol Metab.

[9] Lee SH, Lee WS, Whang KC, Lee SJ, Chung JB (1988). Hyperpigmentation in megaloblastic anemia. Int J Dermatol.

[10] Rzepka Z, Respondek M, Rok J, Beberok A, Ó Proinsias K, Gryko D, Wrześniok D (2018). Vitamin B12 deficiency induces imbalance in melanocytes homeostasis-a cellular basis of Hypocobalaminemia Pigmentary Manifestations. Int J Mol Sci.

[11] Speeckaert R, Van Gele M, Speeckaert MM, Lambert J, van Geel N (2014). The biology of hyperpigmentation syndromes. Pigment Cell Melanoma Res.

[12] Remacha AF, Souto JC, Piñana JL (2011). Vitamin B12 deficiency, hyperhomocysteinemia and thrombosis: a case and control study. Int J Hematol.

[13] Pruthi RK, Tefferi A (1994). Pernicious anemia revisited. Mayo Clin Proc.

[14] Hunt A, Harrington D, Robinson S (2014). Vitamin B12 deficiency. BMJ.

